# VariantSpark: population scale clustering of genotype information

**DOI:** 10.1186/s12864-015-2269-7

**Published:** 2015-12-10

**Authors:** Aidan R. O’Brien, Neil F. W. Saunders, Yi Guo, Fabian A. Buske, Rodney J. Scott, Denis C. Bauer

**Affiliations:** CSIRO, Health & Biosecurity Flagship, 11 Julius Av, Sydney, 2113 Australia; School of Biomedical Sciences and Pharmacy, Faculty of Health, Newcastle, 2308 Australia; Cancer Epigenetics Program, Cancer Research Division, Kinghorn Cancer Centre, Garvan Institute of Medical Research, 384 Victoria St, Sydney, 2010 Australia; UNSW Medicine, University of New South Wales, Sydney, 2052 Australia; CSIRO, Data61, Sydney, 2052 Australia

**Keywords:** Genotype clustering, SPARK, BigData, 1000 Genomes Project, Personal Genome Project, Population structure

## Abstract

**Background:**

Genomic information is increasingly used in medical practice giving rise to the need for efficient analysis methodology able to cope with thousands of individuals and millions of variants. The widely used Hadoop MapReduce architecture and associated machine learning library, Mahout, provide the means for tackling computationally challenging tasks. However, many genomic analyses do not fit the Map-Reduce paradigm. We therefore utilise the recently developed Spark engine, along with its associated machine learning library, MLlib, which offers more flexibility in the parallelisation of population-scale bioinformatics tasks. The resulting tool, VariantSpark provides an interface from MLlib to the standard variant format (VCF), offers seamless genome-wide sampling of variants and provides a pipeline for visualising results.

**Results:**

To demonstrate the capabilities of VariantSpark, we clustered more than 3,000 individuals with 80 Million variants each to determine the population structure in the dataset. VariantSpark is 80 % faster than the Spark-based genome clustering approach, adam, the comparable implementation using Hadoop/Mahout, as well as Admixture, a commonly used tool for determining individual ancestries. It is over 90 % faster than traditional implementations using R and Python.

**Conclusion:**

The benefits of speed, resource consumption and scalability enables VariantSpark to open up the usage of advanced, efficient machine learning algorithms to genomic data.

**Electronic supplementary material:**

The online version of this article (doi:10.1186/s12864-015-2269-7) contains supplementary material, which is available to authorized users.

## Background

Genomic information is increasingly used in medical practice. A commonly performed task in such applications is grouping individuals based on their genomic profile to identify population association [1] or elucidate haplotype involvement in diseases susceptibility [2]. The commonly used tool is ADMIXTURE [3], which performs maximum likelihood estimation of individual ancestries from multilocus SNP genotype datasets.

Due to the decreasing sequencing cost it is now economical to generate studies with sample sizes previously reserved for larger consortia such as the 1000 Genomes Project [4] or The Cancer Genome Atlas, TCGA [5]. At the same time, whole genome sequencing enables the inclusion of rare or even somatic mutations in the analysis, increasing the feature space by orders of magnitude. This drastic increase in both sample numbers and features per sample requires a massively parallel approach to data processing [6]. Traditional parallelisation strategies like MPI/OpenMP or hardware accelerators (GPGPU) cannot scale with variable data sizes at runtime [7] or require purpose-built hardware.

Addressing this issue, APACHE HADOOP MAPREDUCE [8] transforms data into ‘key-value pairs’ that can then be distributed between multiple nodes across a commodity computer cluster, depending on the size of the problem. MapReduce approaches are increasingly being used in bioinformatics (for reviews see [9–11]). This is especially the case for sequence analysis tasks, such as read mapping [12], duplicate removal [13], and variant calling [14, 15] as well as Genome Wide Analysis Study based tasks [16, 17]. Apache has also developed a machine learning library, Mahout [18], which allows efficient out-of-the-box analysis to be applied to clinical applications, such as medical health records [19]. Unfortunately, the MapReduce paradigm is not always the optimal solution, specifically for bioinformatics or machine learning applications that require iterative in-memory computation. Specifically, Hadoop is relying extensively on hard disk input-output operations (disk IO), and this can prove to be a bottleneck in processing-speed.

APACHE SPARK [20] is a more recent compute engine, which overcomes many of Hadoop’s limitations. One of the main benefits is that it allows programs to cache data in memory; potentially eliminating, or at least reducing, the bottleneck of disk IO. When utilising caching, Apache claim SPARK to be up to 100x faster than Hadoop. Although SPARK allows MapReduce-like programs, it does not require programs to exactly model the MapReduce paradigm, which in turn allows more flexible software design. Recognising the capability, Wiewiórka et al. [21] developed SPARKSEQ for high-throughput sequence data analysis and the Big Data Genomics (BDG) group recently demonstrated the strength of SPARK in a genomic clustering application using ADAM, a set of formats and APIs as well as processing stage implementations for genomic data [22]. ADAM is expected to be one of the cornerstones of the Precision Medicine Initiative and the Global Alliance for Genomics and Health [23]. While the speedup of ADAM over traditional methods was impressive (50 fold speedup), being limited by constraints within this general genomics framework can hamper performance.

We hence developed a purpose-built approach in SPARK to perform machine learning tasks on genomic data, such as clustering of individual genomes. We utilise SPARK’s machine learning library, MLlib, and provide an interface to the standard variant data format, Variant Call Format (VCF) [4], which opens up the application of MLlib’s different machine learning algorithms to a wide range of genotype-based analysis tasks.

To demonstrate VARIANTSPARK’s capability, we cluster variant datasets from the 1000 Genomes Project [4] to determine population structure using the *k*-means clustering algorithm available in MLlib. In the first section we benchmark VARIANTSPARK’s performance and accuracy against ADAM and a Hadoop Mahout implementation as well as more traditional methods (R, Python) and the purpose-build tool ADMIXTURE [3]. In the second section we discuss the pipeline for visualising the resulting cluster. In section three we demonstrate VARIANTSPARK’s full capacity by seamlessly scaling from 20 % to 100 % of the human genome. In the last section we replicate the analysis by using 478 genomes from the Personal Genome Project [24].

## Results and discussions

### SPARK enables faster clustering of individuals compared to traditional methods

In this section we compare the time required to cluster individuals based on genomic variants using VARIANTSPARK against ADAM and the more traditional approaches using Hadoop (and Mahout), Python and R, as well as the purpose-build tool ADMIXTURE. We limit the genomic variants to only chromosome 22 as the traditional approaches have substantially larger memory consumption, rendering a whole-genome input infeasible. Furthermore, we perform the comparisons on a single virtual machine to ensure the six different approaches have access to the same resources. We use *k*-means clustering algorithms in the respective implementations, which require the VCF input files to be pre-processed (see methods). The state-of-the-art tool, ADMIXTURE, also requires a pre-processing step from VCF to PED format, which we performed using GATK [15]. We therefore compare the time required for the pre-processing step, as well as for *k*-means clustering.

**Fig. 1:**
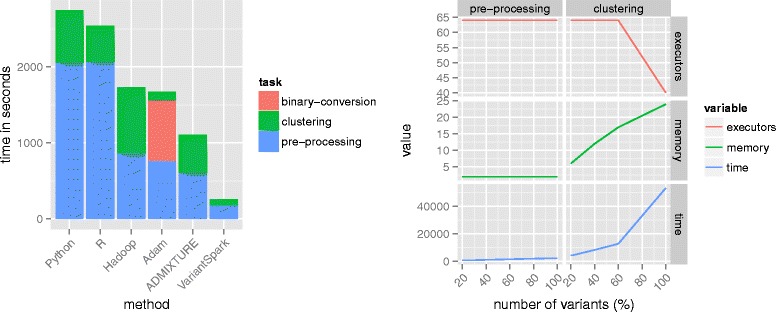
Comparison of method and genome-wide scaling experiment. *Left* Runtime for clustering variants from chromosome 22 is given in seconds with 32 GB of memory on 8 threads (except for the pre-processing in R and Python where this was not supported). *Right* Scaling from 20 % to 100 % of variants in the genome with maximal number of executors and lowest possible memory assignment

As shown in Table 1, pre-processing the data is fastest in VARIANTSPARK, requiring 3 min. This is almost 80 % faster than both the ADAM implementation (13 min) and our Hadoop implementation (14 min). Unlike VARIANTSPARK and our Hadoop implementation, the ADAM framework cannot process the VCF files directly but requires them to be converted into a binary ADAM file format. Although this additional pre-processing step is only required once for each input file, it uses an additional 13 min, rendering our approach almost an order of magnitude faster (see Fig. 1). R and Python are also slower at pre-processing, each taking approximately 34 min. This increased pre-processing time is due to the standard Python and R implementations not natively supporting multithreading, while VARIANTSPARK, ADAM and Hadoop can use the eight available cores. The GATK-based VCF to PED conversion for ADMIXTURE is also slower, with a runtime of over 10 min.

**Table 1:** The resource consumption of the six compared methods as well as the accuracy measured as adjusted Rand index on chromosome 22

Tool	Pre-processing			Clustering			Accuracy
	Threads	Memory	Time	Threads	Memory	Time	
VariantSpark	8	32	2 min 58 sec	8	32	1 min 20 sec	0.84
ADAM	8	32	12 min 48 sec	8	32	1 min 52 sec	0.84
Hadoop	8	32	14 min 22 sec	8	32	14 min 23 sec	0.84
R	1	32	34 min 30 sec	8	32	7 min 25 sec	0.84
Python	1	32	34 min 15 sec	8	32	11 min 29 sec	0.84
Admixture	1	32	10 min 08 sec	8	32	8 min 19 sec	0.25

Clustering the samples is also fastest in VARIANTSPARK (1 min and 20 sec, see Table 1), despite VARIANTSPARK and ADAM both using SPARK’s MLlib *k*-means implementation. We attribute the 30 % speedup over ADAM (1 min and 52 sec) to VARIANTSPARK converting the VCF files to sparse vectors, whereas ADAM creates dense vectors, which are less memory efficient. Multithreading *k*-means clustering is natively supported by both R and Python, we are therefore able to utilise eight cores. Despite this, both are an order of magnitude slower than VARIANTSPARK and ADAM, with Python requiring 11 min and R requiring 8 min. ADMIXTURE is also slower with a runtime of just over 8 min. Our Hadoop implementation performs the worst, at 14 min, showcasing the limitations of the Hadoop engine for iterative algorithms such as *k*-means. Hadoop writes the entire output of each *k*-means iteration to disk. This feature enables scalability but, in this case, substantially increases the runtime as disk IO becomes the limiting factor for this small dataset (chromosome 22). Python and R store the output from each iteration in memory, thereby eliminating the IO bottleneck, but without being able to utilise disk storage they are limited to datasets that fit into memory. SPARK overcomes both limitations by performing in-memory caching for each executor, which eliminates the disk IO bottleneck on smaller datasets, while maintaining scalability to larger datasets by spilling data that exceeds memory availability to disk.

We also investigate the cluster quality for the five different methods by comparing the annotated super-population label (AMR, AFR, EAS, SAS) for each individual in the 1000 Genomes data to the label assigned by *k*-means clustering. For this comparison, we use the adjusted Rand index (ARI) metric, which returns a value between -1 (independent labelings) and 1 (perfect match) [25].

Using chromosome 22, each of the algorithms resulted in an ARI of 0.84, confirming that the speed-up in SPARK is not at the cost of quality. The state-of-the-art tool, ADMIXTURE, returns a low ARI of 0.25. It should be noted that ADMIXTURE’s underlying statistical model does not take linkage disequilibrium (LD) into account. Therefore removing variants with high LD may result in higher accuracy. We can substantially improve the accuracy to a perfect classification (ARI=1.0) by removing the fourth super-population, AMR (American). This is due to the majority of AMR individuals being placed in the same group as Europeans, likely reflecting their migrational backgrounds. Only a minority of AMR individuals form an independent group, likely comprising of genetic information otherwise not captured by the 26 sub-populations of the 1000 Genomes Project. This indicates that although allelic differences exist between populations, “genetic diversity is distributed on a continuum” [26].

### Graph visualisation

**Fig. 2:**
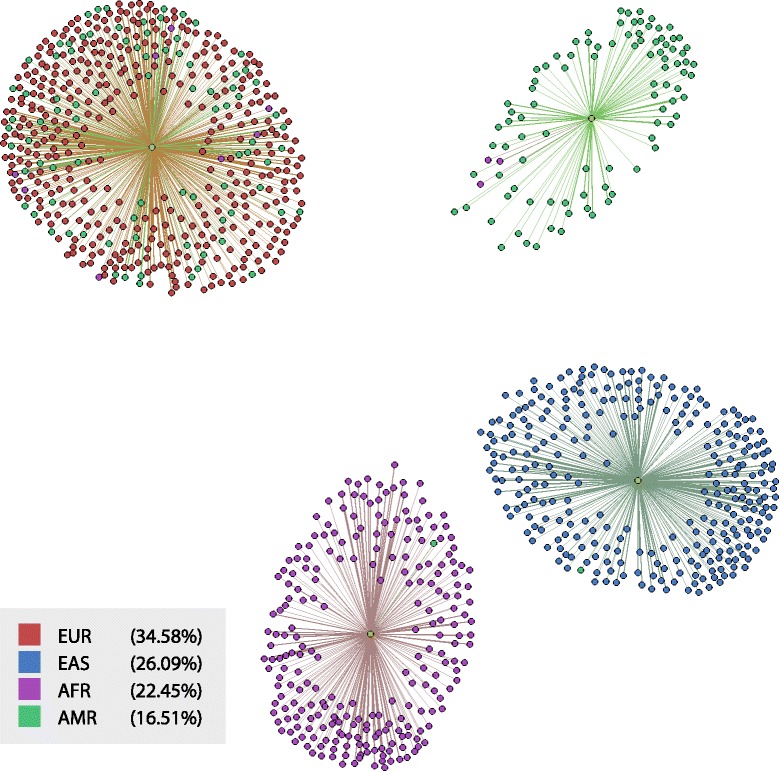
Visualisation of VARIANTSPARK predicted clusters. The figure shows the four clusters predicted for the 1000 Genomes data. Individuals from the super-populations AFR, AMR and EAS are accurately grouped into distinct clusters. The fourth cluster contains predominantly EUR + AMR individuals potentially accurately reflecting migrational backgrounds

VARIANTSPARK supports visualisation of the resulting population clusters by generating a file that can be loaded into GEPHI [27]. Figure 2 visualises the clusters generated by VARIANTSPARK from the 1000 Genomes Project data. As discussed in the previous section, three of the four clusters (super-populations AFR, EAS and AMR) are relatively homogeneous; the fourth is more mixed and consists predominantly of individuals labelled EUR and AMR providing a visual representation of the ARI of 0.84.

### VARIANTSPARK enables genome wide sampling of variants to improve clustering quality

Although we have demonstrated the advantages of VARIANTSPARK over traditional methods on small datasets, such as individual chromosomes, the main advantage of SPARK is its ability to process datasets that exceed memory limits. To demonstrate this scalability, we increase the number of variants used in the clustering from the initial 494,328 on chromosome 22 to 38,219,238 variants across the 1000 Genomes Project dataset (phase 1).

We run this comparison on our in-house HADOOP/SPARK cluster using 40 cores. Pre-processing the VCF files takes approximately 12, 19, 27 and 40 min for 20, 40, 60 and 100 percent of the genome, respectively (see Table 2). In each case, the memory required does not exceed a modest 2 GB per executor, even for the complete genome. Clustering the data takes 70, 139, 213 and 884 min, respectively. For clustering, the memory requirements increase approximately linearly to 24 GB per executor (see Fig. 1). The increase in memory is due to more distance measurements between variants and *k*-means centroids being created, which MLlib stores as dense vectors. While *k*-means clustering can scale to 100 % of the genome, machine learning algorithms that can deal with sparse vectors would potentially be able to scale much further and potentially process more alleles.

**Table 2:** The resources consumption on different subsets of the entire autosome (chromosomes 1–22) of phase 1 as well as all of phase 3. Memory specified is the memory allocated to each executor

Data	Portion	Pre-processing			Clustering		
		Executors	Memory	Time	Executors	Memory	Time
Phase 1	20 %	64	2	11 min 53 sec	64	6	1 h 10 min
	40 %	64	2	19 min 09 sec	64	12	2 h 19 min
	60 %	64	2	26 min 34 sec	64	17	3 h 33 min
	100 %	64	2	40 min 48 sec	40	24	14 h 44 min
Phase 3	100 %	64	2	3 h 54 min 24 sec	40	24	27 h 46 min

We do not observe an increase in cluster accuracy when providing variants from all chromosomes, indicating that current practice of clustering based on a subset of the genome is sufficient to define the existing population boundaries for populations with large genetic distance [26].

To further demonstrate the scalability of VARIANTSPARK, we also cluster the 1000 Genomes Project phase 3 data, which contains 3000 individuals from 5 super-populations and as a result has over 80 Million variants. The uncompressed size of the phase 3 files is 770 GB compared to 161 GB for the phase 1 dataset. VARIANTSPARK successfully completes the clustering in 30 h (see Table 2) with an ARI of 0.82.

### Determining the population structure of the Personal Genome Project

To demonstrate the versatility of VARIANTSPARK we also process and cluster individuals from the Personal Genomics Project (PGP). The PGP is open to the public for individuals to submit their genomic sequence along with any metadata, such as diseases or clinical features [24]. We obtain and curate the data (see methods) resulting in 985,790 variants from 478 individuals.

We cluster the individuals allowing five clusters and compare the assigned labels to the same number of self-reported ‘Race/ethnicity’ labels (‘White’, ‘Hispanic or Latino’, ‘Asian’, ‘Black or African American’ and ‘American Indian’). The observed ARI is negative, indicating the clustering is random. This poor result may be due to the labels being too broad and not accurately reflecting genetic diversity. For example, the ‘White’ label includes individuals with grandparents from United States, Syria, Arab Republic, or Bulgaria, amongst others. The dataset is also very one-sided with 433 out of the 478 (over 90 %) of the individuals labeled as white, even though geographical location of their grandparents are very diverse. We therefore only include individuals where all grandparents were reported to be from the same country leaving 35 individuals from 22 different countries, which we group into their approximate super-populations (see Additional file 1: Table S1). Clustering these individuals results in an ARI of 0.45.

Although we see an increase in cluster-accuracy when removing individuals with a mixed background and operating at super-population level, the clustering is not as precise as on the carefully curated and characterised individuals from the 1000 Genomes Project. This is especially the case since 65 % are ‘White’ Americans, which formed the group most difficult to cluster in the 1000 Genomes Project data.

This highlights the issue for clinical application where ancestry influences treatment (e.g. HLA allele genotyping from SNP information [28]) and accurate population association may not be known for patients with diverse migrational background. As noted by Patterson et al. [29], more markers are needed for populations with low genetic divergence. It is hence likely that higher density genotyping (e.g. from genome sequencing) would help in elucidating population structure in this dataset, which demonstrates the need for fast whole-genome approaches. Unfortunately, this hypothesis can not be tested as whole genome information is not available for these individuals.

## Conclusions

VARIANTSPARK performs clustering on VCF files with over 3000 individuals and 80 million variants in 30 h using minimal memory (24 GB). VARIANTSPARK supports random genome-wide sampling of variants allowing faster clustering for well characterised cohorts where 20 % of the genome is sufficient. On the benchmarking dataset, it outperforms ADAM by almost an order of magnitude (4 vs 28 min) by processing VCF files directly and storing the information in sparse vectors. VARIANTSPARK utilises SPARK, which allows for in-memory caching and hence performs 86 % faster than Hadoop (29 min). VARIANTSPARK scales to data sizes that are not feasible to process using R or Python due their requirement to load the whole dataset into memory. But even on the small benchmarking dataset, VARIANTSPARK’s novel parallelisation approach is faster than traditional multithreading with 90 % speedup over R (42 min), and 91 % over Python (45 min). VARIANTSPARK is also superior in performance and accuracy over the current state-of-the-art tool for individual ancestries determination, ADMIXTURE. These benefits of speed, resource consumption and scalability allow VARIANTSPARK to be the interface for applying other machine learning tools from MlLib to genomic data. Utilising MLlib as well as the more recent addition, SparkML, will enable supervised machine learning applications to e.g. identify variants that jointly interact with phenotypes as well as include electronic health record in addition to the genomic feature vector to e.g. capture medical history as well as predispositions for diagnosis and treatment decisions.

## Implementation

### Computational resources

We completed the Chromosome 22 comparisons on a virtual machine (VM) hosted on Microsoft Azure. This VM is an A7 Linux instance with 8 cores, 56 GB memory running Ubuntu. For the whole-genome clustering, we used our in-house Hadoop cluster with Hadoop 2.5.0, managed by Cloudera’s CDH 5. We use Spark 1.3.1. This 13 node cluster has a total of 416 cores and 1.22 TB memory.

### Datasets

We use the 22 autosomes from the 1000 Genomes Project phase 1 dataset. This dataset contains variants from 1092 individuals, across 38,219,238 alleles. For the smaller dataset, we use the variants from chromosome 22, with 494,328 alleles. The individuals from these datasets are distributed across four super populations, African (AFR), Mixed American (AMR), East Asian (EAS) and European (EUR). We also cluster the Phase 3 dataset, which contains variants from 2535 individuals, across 81,271,745 variants. As well as the above for super populations, this dataset also includes the South Asian (SAS) super population.

### VariantSpark implementation

To cluster individuals from a VCF file, we initially need to pre-process the variants to feature vectors. In VARIANTSPARK we read in VCF files as text files to a Resilient Distributed Dataset (RDD). RDDs allow us to process the files, line-by-line, in parallel.

**Fig. 3:**
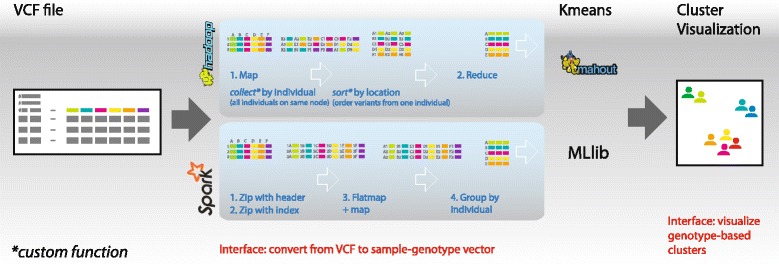
Schematic overview of VariantSpark. The image shows the flow from the input VCF file to the machine learning library and onto the visualization. It highlights the differences between the Hadoop and Spark implementations for converting data in VCF format to a data structure readable by Mahout and MLlib, respectively

We parse each line as tab-separated values, and store the values from each line in an array. For each array, we use SPARK’s ‘zip’ function to create tuples of the values with their respective heading (sample name). Now each array element is a key-value pair (KVP) of the previous value and its heading. For the KVPs that contain an allele as the value, the key (derived from the heading) is the individual ID (see Fig. 3, Spark step 1).

For each array, we remove any KVPs that represent data other than alleles (such as the genomic location). With the remaining KVPs, which all represent alleles, we apply a function to convert these from the strings found in VCF files (i.e. ‘0|1’), to doubles, where the double is the Hamming distance to the reference. I.e. a 1 represents a heterozygous variant, 2, a homozygous variant, and 0, no variant. Because we will eventually be converting this data to sparse vectors, we remove any KVPs with a value of 0 (i.e. no variant).

At this stage, we can optionally filter variants that do not match a specific criteria. For the Phase 3 dataset, we exclude rare variants that are present in only one individual. Because each array now only contains variants (rather than every allele), we can simply drop arrays with a length of one, as the length of each array is equal to the number of individuals who have a variant at that allele.

Now that the dataset only contains alleles we are interested in, we zip each array with a unique sequential index (allele ID). This index will serve as identifier for each allele (and keep the genome loci order) (step 2).

Although we now have the data we require, the variants are stored in arrays of alleles, whereas we require the data to be transposed into arrays of individuals. To facilitate the grouping of variants by individuals, we use ‘flatMap’ to flatten out the arrays into a collection of KVPs, where the key is the individual ID, and the value is a tuple of the variant ID and the variant.

We can now group these KVPs by their key (individual ID) and save them to sparse vectors (step 4).

### ADAM implementation

For our ADAM comparison, we followed the ADAM implementation from (http://bdgenomics.org/blog/2015/02/02/scalable-genomes-clustering-with-adam-and-spark/).

### Hadoop implementation

Our Hadoop implementation is based on the MapReduce model, which utilises KVPs, similarly to VARIANTSPARK. As we need a unique range of identifiers for alleles (in the smallest range possible), we need to run an initial MapReduce task to index the lines. This is comparable (however, more verbose) to the ‘zip’ operation in VARIANTSPARK. The second MapReduce task does the bulk of the work and is visualised in Fig. 3. The Map stage (step 1) begins by creating KVPs from the VCF file. For each KVP, the key is a tuple of a primary and secondary key, where the primary key is the individual ID and the secondary key is the allele ID. The value for each KVP is the variant. The primary key ensures that KVPs for each individual are distributed to the same node during the MapReduce shuffle stage. After being distributed, the KVPs for each individual are sorted by their secondary key. Now that the KVPs for each individual are physically located on the same hardware, the Reduce stage (step 2) can efficiently create a sparse vector for each individual from these KVPs.

### R implementation

We utilise READGT from the VARIANTANNOTATION package for reading in the VCF file and extracting the genotype matrix. In the Additional file 1 we demonstrate that this approach is approximately one minute faster than using R’s built-in READ.TABLE function.

As with our VARIANTSPARK pre-processing, we convert the strings that represent each allele to a numeric value. This process consists of applying our HAMMING function to the dataframe with SAPPLY. We then transpose the matrix with T(VCFMATRIX), which results in a data-structure where each row represents an individual. We convert the matrix to a BIG.MATRIX object, as required by the *k*-means algorithm from the ‘biganalytics’ package (https://cran.r-project.org/web/packages/biganalytics/index.html), and then call BIGKMEANS with the BIG.MATRIX object and the required number of clusters as arguments. See git repository for more details.

### Python implementation

Our Python implementation reads in lines from a VCF file as tab-separated values using DATAFRAME.READ_CSV, and stores the data in a pandas DataFrame (http://pandas.pydata.org). The column headings are the individual IDs and the row headings are the allele locations. We remove the first 9 columns and convert the remaining allele strings to numeric values by applying our HAMMING function to the DataFrame. We then convert the DataFrame to a matrix with .AS_MATRIX() and cluster the matrix using ‘scikit-learn’ (http://scikit-learn.org/stable/). See git repository for more details.

### ADMIXTURE implementation

We use Genome Analysis Toolkit (-T VARIANTSTOBINARYPED) [15] to convert the variants from VCF, and a 1000 Genomes Project supplied.ped file, to a binary PLINK (.bed), binary marker information file (.bim) and pedigree stub file (.fam). These three files are used as input to ADMIXTURE, with default options and *K* (the number of ancestral populations) set to 4.

### Personal genome project data

We acquired genomic data from the Personal Genome Project (PGP) website (https://my.pgp-hms.org/public_genetic_data). We sort the genotype data from 23andMe microarray platforms by genome build. Using a custom shell script, we convert NCBI build 36 files to BED format, update to build 37 using UCSC liftOver (https://genome.ucsc.edu/cgi-bin/hgLiftOver) and then convert back to 23andMe format. We then convert the 23andMe files to VCF using code obtained from the Broad Institute (http://apol1.blogspot.com.au/2013/08/impute-apoe-and-apol1-with-23andme.html). Where individuals had genotype data for both genome builds 36 and 37, we only use the latter. Finally, we combined the individual VCF files into one file for clustering, using VCFtools [30].

## Availability

The package is written in Scala and available at https://github.com/BauerLab/VariantSpark.

## Additional file

Additional file 1
**PGP population labels and implementation details.** Conversion map self-reported descriptive population labels to super-population labels. Details of the R implementation. (PDF 22.2 kb)
