# Supplementary material for: VariantSpark: population scale clustering of genotype information
Source: BMC Genomics. 2015 Dec 10;16:1052. doi: 10.1186/s12864-015-2269-7 (PMC4676146; doi:10.1186/s12864-015-2269-7)
Supplement: Additional file 1 — PGP population labels and implementation details. Conversion map self-reported descriptive population labels to super-population labels. Details of the R implementation. (PDF 22.2 kb) [file 12864_2015_2269_MOESM1_ESM.pdf]

Table S1 Country of origin assignment to super-population.

Super Population	Super Population Size	Populations	Population size
AMR	5	Canada	2
		Mexico	1
		Puerto Rico	1
		US Minor Outlying Islands	1
EAS	6	China	5
		Taiwan, Province of China	1
EUR	20	Belgium	1
		Estonia	1
		Finland	1
		France	1
		Germany	1
		Greece	1
		Ireland	3
		Poland	2
		Russian Federation	1
		Slovenia	1
		Spain	2
		Sweden	1
		Ukraine	2
		United Kingdom	2
SAS	4	Bangladesh	1
		India	3

1 R implementation comparison

We compare our implementation using READ.TABLE to one using the 'VariantAnnotation' package. Reading the VCF file to a matrix with 'readGT' is faster than reading a VCF file to a table using R's 'read.table', at 138 seconds compared to 208 seconds. However, the entire process takes around 30 minutes, so the relative savings in time are minimal. One other difference is that 'readGT' produces a matrix of variants, so we don't need to remove the first nine columns. However, this step takes less than one second. The function to convert the variant strings to the Hamming distance consumes the majority of the time required for preprocessing. However, we created this as a vectorized function to improve performance.
